# Enhanced Fault Diagnosis in Milling Machines Using CWT Image Augmentation and Ant Colony Optimized AlexNet

**DOI:** 10.3390/s24237466

**Published:** 2024-11-22

**Authors:** Niamat Ullah, Muhammad Umar, Jae-Young Kim, Jong-Myon Kim

**Affiliations:** PD Technology Co., Ltd., Ulsan 44610, Republic of Korea; niamatktk@gmail.com (N.U.); umarengineer499@gmail.com (M.U.); kjy7097@pdtech.co.kr (J.-Y.K.)

**Keywords:** milling machine, feature optimization, fault diagnosis, modified AlexNet, support vector machine, ant colony optimization

## Abstract

A method is proposed for fault classification in milling machines using advanced image processing and machine learning. First, raw data are obtained from real-world industries, representing various fault types (tool, bearing, and gear faults) and normal conditions. These data are converted into two-dimensional continuous wavelet transform (CWT) images for superior time-frequency localization. The images are then augmented to increase dataset diversity using techniques such as rotating, scaling, and flipping. A contrast enhancement filter is applied to highlight key features, thereby improving the model’s learning and fault detection capability. The enhanced images are fed into a modified AlexNet model with three residual blocks to efficiently extract both spatial and temporal features from the CWT images. The modified AlexNet architecture is particularly well-suited to identifying complex patterns associated with different fault types. The deep features are optimized using ant colony optimization to reduce dimensionality while preserving relevant information, ensuring effective feature representation. These optimized features are then classified using a support vector machine, effectively distinguishing between fault types and normal conditions with high accuracy. The proposed method provides significant improvements in fault classification while outperforming state-of-the-art methods. It is thus a promising solution for industrial fault diagnosis and has potential for broader applications in predictive maintenance.

## 1. Introduction

In modern manufacturing, ensuring the reliability and efficiency of milling machines is critical owing to their extensive use in many industries, such as aerospace [1], automotive [2], and precision engineering [3,4]. Milling machines transform raw materials into finished products by executing complex operations, such as cutting, grinding, and drilling, at high speeds and under significant mechanical loads [5]. These operating conditions make milling machines susceptible to faults, particularly in important components like cutting tools, bearings, and gears. Mechanical faults, which account for 57% of all failures in milling machines, can lead to considerable downtime and financial losses. Among these faults, bearing failures represent 42% of the issues, while tool-related defects account for nearly 20% of unplanned downtime. These faults not only increase operational costs but also impact production timelines, making early detection essential for maintaining high productivity levels. This study therefore focused on the development of an advanced fault diagnosis system that detects mechanical component failures in milling machines, particularly those involving bearings, gears, and cutting tools [6].

Over the past decade, advancements in machine learning, feature engineering, and deep learning have significantly enhanced condition monitoring systems. Recent studies have demonstrated their ability to detect faults reliably under complex operating conditions. For instance, one study demonstrated a machine-learning approach for fault detection in robotic systems using electrical current signatures [7]. Raouf et al. [8] highlighted the benefits of transfer learning in adapting models for different industrial systems. These studies underscore the growing importance of combining domain knowledge with modern AI techniques for fault detection [9]. Furthermore, the emergence of explainable artificial intelligence (XAI) methods, such as the work on polymer composite structures, has emphasized the need for interpretable models in industrial applications [10]. These advancements serve as a foundation for our study, which helps AE-based monitoring for fault diagnosis. Condition monitoring involves real-time data collection from machines through sensors, followed by processing the data using AI algorithms to predict or detect faults before they lead to significant failures [11]. The literature identifies two main approaches to condition monitoring: direct methods, which focus on physical changes in the machine, and indirect methods, which analyze signals generated by the machine during operation [12]. While direct methods, such as visual inspections or wear measurements, offer high accuracy, they are often invasive, time-consuming, and sensitive to environmental factors such as lighting and fluid exposure. Indirect methods, on the other hand, utilize sensors to capture signals, such as vibrations or acoustic emissions (AEs), which are then processed through machine learning models for fault detection. AE-based monitoring has become increasingly popular owing to its high sensitivity to fault-related events and its ability to capture signals in noisy environments. AEs operate at frequencies higher than typical machine vibrations. They consequently reduce interference from ambient noise, which makes them suitable for monitoring components such as bearings and cutting tools in milling machines.

AEs are generated as a result of shear stresses between the tool and workpiece during machining. Assessing AEs can elucidate the changes in material removal, tool wear, and gear faults [13]. These signals can be collected using AE sensors, which are relatively easy to install and maintain. Compared to traditional monitoring methods, AEs offer significant advantages in precision, particularly in the detection of early-stage faults in rotating machinery. This study thus leveraged AE-based monitoring to detect faults in fundamental milling machine components, including bearings, gears, and cutting tools, using deep learning-based methods.

### Related Literature

AE refers to the release of elastic energy from materials during deformation or fracture. AEs are detected by sensors as acoustic emission hits (AEHs). Faults in milling machines alter the distribution of AE signals. This has prompted researchers to explore various methods for extracting fault-related features from these signals in the temporal domain (TD), spectral domain (SD), and time-frequency domain (TFD) [14,15,16]. Once features are extracted, machine-learning models, particularly AI-based techniques, can be utilized to identify and classify faults [17].

In earlier work, Twardowski et al. [18] developed a framework using time-domain indicators, such as root mean square (RMS) values, to classify faults using a decision tree model. Shao et al. explored how convolutional neural networks (CNNs) can be used to diagnose induction motor faults by directly learning features from vibration signals. Medina et al. [19] employed AE Poincaré plots and random forest classifiers to detect gear defects such as broken teeth and scuffing. Li et al. [20] used long short-term memory (LSTM) and support vector data description (SVDD) to monitor tool wear based on one-dimensional TD signals [21]. While TD analysis is valuable, spectral domain analysis is particularly useful in diagnosing faults, as faults in machine components often alter the frequency spectrum of AE signals. Bai et al. [22] proposed a lightweight deep learning model that outperformed traditional neural network classifiers when trained on a combination of TD and SD indicators. Wang et al. [23] employed empirical mode decomposition (EMD) for signal preprocessing, followed by random forest classification for detecting faults in tools. However, owing to the non-stationary nature of AE signals from milling machines, advanced TFD techniques, such as wavelet transform (WT), EMD, and feature mode decomposition (FMD), are often necessary for effective fault diagnosis [24,25].

Several studies have leveraged these advanced techniques to enhance fault detection. Hussain et al. [26] used wavelet-decomposed signals to extract statistical indicators, such as the mean, standard deviation, and entropy, for fault diagnosis in machining centers. Ding et al. [27] proposed a model combining discrete WT and lightweight CNNs to identify faults in spindles and tools. Wang et al. [28] utilized EMD to develop a tool fault diagnosis framework, which includes random forest classification of features extracted from decomposed AE signals.

Despite their success, the above techniques also have limitations. TD indicators, while sensitive to noise, may lack robustness in diagnosing complex faults. SD methods are suitable for stationary signals; however, they struggle with the non-stationary nature of AE signals from milling machines. Although TFD techniques, such as EMD, are more effective, they are computationally costly and often require expert domain knowledge for fine-tuning parameters such as filter length and mode numbers. To address these challenges, this study designed an enhanced approach for fault diagnosis using advanced deep learning architectures.

AEH events result from hazardous occurrences such as material fractures or stress cracks during milling operations, generating transient acoustic waves detected by AE sensors. When faults develop, these stress waves induce distinct transients in AE signals, commonly referred to as AEHs or events. Extracting these fault-related features from AE signals is vital for diagnosing faults in milling machines. By analyzing these features, it becomes possible to classify machine health based on the patterns detected in the AE signals. However, isolating these burst-like events can be challenging owing to background noise and vibrations within the machine environment. To address these difficulties, this study employed an approach in which AE signals are first converted into continuous wavelet transform scalograms. These scalograms provide a detailed time-frequency representation, capturing both transient and non-stationary fault characteristics [29]. Since noise in AE signals can reduce diagnostic accuracy, an image sharpener is applied to the scalograms. This technique sharpens the edges and enhances useful features while maintaining important fault-related data. It improves the visibility of the distinct features, ensuring that the feature extraction process is grounded in clean and reliable data.

Deep learning techniques have gained widespread popularity in recent years owing to their ability to automatically learn and extract meaningful features from raw data without manual intervention [30]. CNNs are highly effective in extracting spatial features from image data and signals [31]. CNNs have demonstrated success in diagnosing mechanical component failures, such as bearing faults, gear defects, and tool wear, by processing AE signals or vibration data. However, CNNs primarily focus on spatial feature extraction and may struggle to capture the temporal dependencies inherent in sequential AE signal data.

Fault detection in milling machines presents several challenges due to their complex operational conditions, including high-speed machining, non-stationary signals, and the presence of significant noise. Traditional monitoring methods, such as TD or spectral analysis, often struggle with these challenges because they cannot capture transient and non-linear features in noisy environments. Furthermore, the non-stationary nature of AE signals makes it difficult to accurately diagnose faults, particularly for early-stage defects in components like bearings, gears, and cutting tools. To address this limitation, we propose a variant of AlexNet 3D, which incorporates three residual blocks to improve the network’s ability to capture both spatial and temporal features. The AlexNet 3D architecture extends the traditional AlexNet model by combining 3D convolutions, which enable the extraction of spatiotemporal features from AE signals. The inclusion of residual blocks helps address the vanishing gradient problem commonly faced in deep networks, allowing for better gradient flow and improved training performance. These residual blocks enhance the model’s depth without compromising its ability to learn effective representations of the input data. By leveraging AlexNet 3D and its residual blocks, this study strived to improve the fault classification accuracy of milling machines by capturing both local and global patterns in AE signals.

Furthermore, deep feature extraction is performed using an average pooling layer to reduce the dimensionality of the feature maps while retaining the most important information. This process enables the model to generate a compact representation of the input data, which can then be used for classification tasks. After feature extraction, the extracted features are fed into a support vector machine (SVM) classifier, which is known for its power in handling high-dimensional data and achieving high classification accuracy. The integration of an SVM ensures a more precise separation between the different fault categories. The model’s ability to reduce the parameter count from 60 million (traditional AlexNet) to 9.8 million (AlexNet 3D with residual blocks) further enhances its computational efficiency, making it suitable for real-time fault diagnosis in industrial settings. This approach enhances the model’s ability to detect faults in milling machine components, including bearings, gears, and cutting tools. Thus, the primary contributions of this study are as follows:

1. A method is proposed for fault classification in milling machines utilizing advanced image processing and deep learning techniques.
2. A modified AlexNet is introduced with three residual blocks to extract spatial and temporal features from augmented scalograms, thereby enhancing feature representation for fault classification.
3. Extracted deep features are optimized using ant colony optimization (ACO) to retain the most relevant information. The optimized features are then classified using an SVM, resulting in highly accurate fault classification.
4. The effectiveness of the proposed methods was evaluated using real-world AE data collected from milling machines.

The remainder of this paper is organized as follows: Section 2 discusses the technical concepts and methodologies used in the study. Section 3 provides an in-depth description of the proposed deep learning model. Section 4 presents the experimental results and evaluation. Section 5 provides conclusions and directions for future research.

## 2. Proposed Work

**Figure 1:**
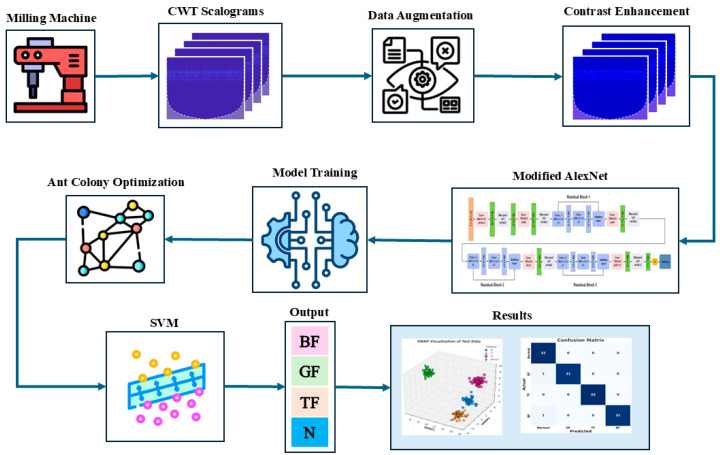
Workflow and overall process of a proposed fault classification method for milling machines.

The complete workflow of the proposed model is shown in Figure 1. The proposed method is outlined as follows.

Step 1: The raw fault data (CWT images representing tool, bearing, and gear faults and normal conditions) are augmented to increase the dataset size and variability. Techniques such as rotating, scaling, and flipping are applied, helping to improve model generalization by learning from diverse examples.
Step 2: A contrast enhancement filter is applied to the augmented images. This step enhances key features in the data, making fault-related patterns more prominent, which in turn improves the ability of the model to effectively learn these features.
Step 3: The enhanced data are then input into a modified AlexNet model. This modification includes the addition of three residual blocks to enhance network efficiency. Unlike traditional models, this architecture extracts local and temporal dependencies from the CWT images. This process is especially important for fault detection, as it allows the model to capture both spatial features (local information) and time-based patterns (temporal information), which are crucial for identifying fault behaviors in milling machines.
Step 4: The modified AlexNet is trained from scratch on augmented, contrast-enhanced data. The network learns to extract deep features that represent both the spatial and temporal characteristics of the different fault types (tool, bearing, and gear faults) and normal conditions. This improves the model’s ability to detect subtle and time-dependent fault signals.
Step 5: After training, the deep features representing both local and temporal dependencies are extracted from the model. These features are rich in information, capturing key patterns over time, and serve as input for further optimization. The feature vector size is 640 × 1000.
Step 6: The extracted features are then optimized using ACO. ACO selects the most relevant features, focusing on those that provide the best distinction between different fault types and reducing the dimensionality of the feature set for improved classification accuracy. The feature vector size is 640 × 800.
Step 7: The optimized features are then fed into an SVM classifier. The SVM uses these optimized features to classify the fault types (tool fault, bearing fault, gear fault, or normal condition). The SVM classifier excels at separating the data into different categories based on the most important features and providing the final fault classification output.

### 2.1. CWT

CWT is a mathematical technique that is used to analyze non-stationary signals, which are signals whose frequency characteristics change over time. Unlike traditional frequency analysis methods, such as the Fourier transform, which decomposes a signal into sinusoidal components, CWT uses wavelets, which are short, oscillating functions localized in both time and frequency. This makes it particularly useful for studying transient and time-varying phenomena in signals. CWT is mathematically defined by Equation (1).

(1) $${CWT}_{x}\left(\tau ,s\right)=\frac{1}{\sqrt{\left|s\right|}}\int_{-\infty }^{+\infty }X\left(T\right)\cdot \psi^{*}\cdot \left(\frac{T-\tau }{s}\right)dt$$

where $X\left(T\right)$ is the signal to be analyzed, and $\psi^{*}$ is the complex conjugate of the mother wavelet, a localized oscillating function chosen based on the type of signal being analyzed. Moreover, s is the scale parameter, which controls the wavelet’s frequency. A smaller scale corresponds to a higher frequency, revealing more finely detailed high-frequency components. A larger scale corresponds to lower-frequency components and broader signal trends. Furthermore, the translation parameter τ shifts the wavelet along the time axis, allowing it to capture localized events at different times [32].

**Figure 2:**
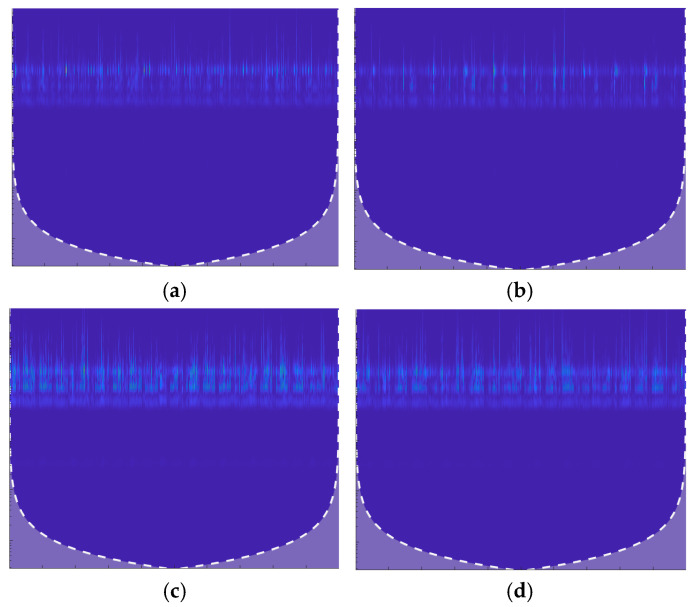
CWT images representing different fault conditions: (**a**) BF; (**b**) GF; (**c**) TF; and (**d**) N.

CWT works by translating and scaling the mother wavelet over the signal. The result is a time-frequency representation that shows how the signal’s frequency content changes over time, as shown in Figure 2. For each combination of scale s and time τ, CWT produces a coefficient that represents the correlation between the signal and wavelet at that specific scale and position. These coefficients can be visualized using a scalogram, which is a 2D plot where the *x*-axis represents the time (τ), and the *y*-axis represents the scale $\left(s\right),$ or, inversely, the frequency. The color intensity represents the wavelet coefficient magnitude, highlighting the energy or power of the signal at specific time and scale points. CWT was chosen for this study due to its superior ability to analyze non-stationary signals like AE signals. Unlike the STFT, which uses a fixed window size, CWT provides multi-scale analysis, enabling the capture of both fine and coarse features in the time-frequency domain. This capability is useful for detecting transient fault characteristics in AE signals.

### 2.2. Contrast Enhancement

**Figure 3:**
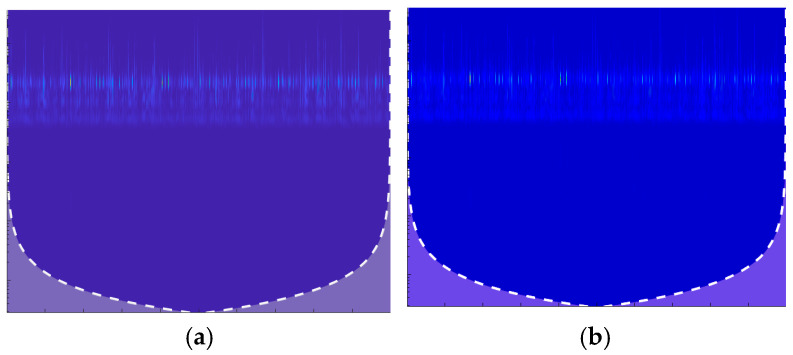
Comparison of CWT images before (**a**) and after (**b**) applying contrast enhancement.

Contrast enhancement is an essential preprocessing step that is applied to the augmented CWT images to improve the visibility of key fault-related features, as depicted in Figure 3. By enhancing the subtle differences between normal and faulty conditions, the model can more effectively differentiate between these states. Techniques such as histogram equalization and adaptive contrast enhancement ensure that important spatial and temporal patterns in the images are more easily detected by the modified AlexNet model. This enhances the model’s ability to learn meaningful features and improves its overall classification performance, making it more effective at detecting tool, bearing, and gear faults.

### 2.3. Modified AlexNet

**Figure 4:**
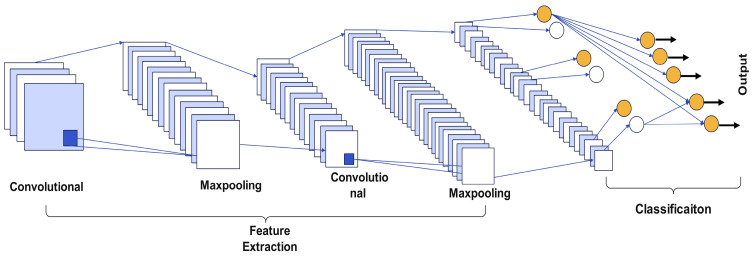
The architecture of the modified AlexNet model for feature extraction.

AlexNet marked a remarkable advancement in adopting GPU acceleration to enhance the performance of CNNs [33]. The CNN architecture, as shown in Figure 4, consists of respective convolutional, max-pooling, normalization, and fully connected layers, and it culminates in a SoftMax layer.

The convolutional layers contain convolutional filters followed by a nonlinear activation function, typically a ReLU layer. Pooling layers are used to perform max pooling, and the input size is fixed owing to the presence of the fully connected layer. In the proposed model, the input size is 227 × 227 × 3.

#### AlexNet Architecture with Residual Blocks and Parameter Reduction

**Figure 5:**
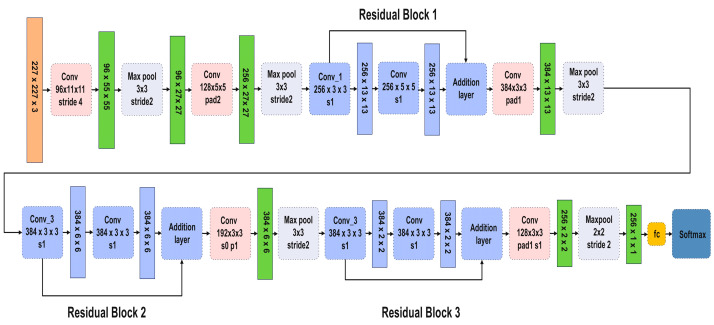
Architecture of the modified AlexNet, featuring three residual blocks for enhanced feature extraction.

The modified AlexNet is introduced with residual blocks that reduce the number of parameters and improve the flow of gradients, making the model easier to train. Each residual block consists of two convolutional layers, two ReLU activations, and one batch normalization layer. These blocks are connected to the original layers of the AlexNet with an additional layer after each residual block, as observed in Figure 5.

In Residual Block 1, the first convolutional layer has 256 filters with a filter size of 3 × 3, a stride of 1 × 1, and padding set to the same. The operation for the first convolutional layer is represented as Equation (2):

(2) $$Y_{1}=Conv2D\left(X,W_{1}\right)+b_{1}$$

where *X* is the input tensor, *W*_1_ represents the filter weights, and *b*_1_ is the bias. After the convolution, batch normalization is applied across the 256 channels to normalize the activations, followed by the ReLU activation function given in Equation (3):

(3) $$A_{1}=ReLU(BN(Y_{1}))$$

Next, the second convolutional layer in this block uses 256 filters with a filter size of 5 × 5, a stride of 1 × 1, and the same padding. The second convolution operation is given in Equation (4):

(4) $$Y_{2}=Conv2D\left(A_{1},W_{2}\right)+b_{2}$$

Again, the output passes through batch normalization and ReLU activation, as in Equation (5):

(5) $$A_{2}=ReLU(BN(Y_{2}))$$

At the end of this block, output *A*_2_ is added to the original input, *X*, creating the residual connection, as in Equation (6):

(6) $$Z_{1}=A_{2}+X$$

**Figure 6:**
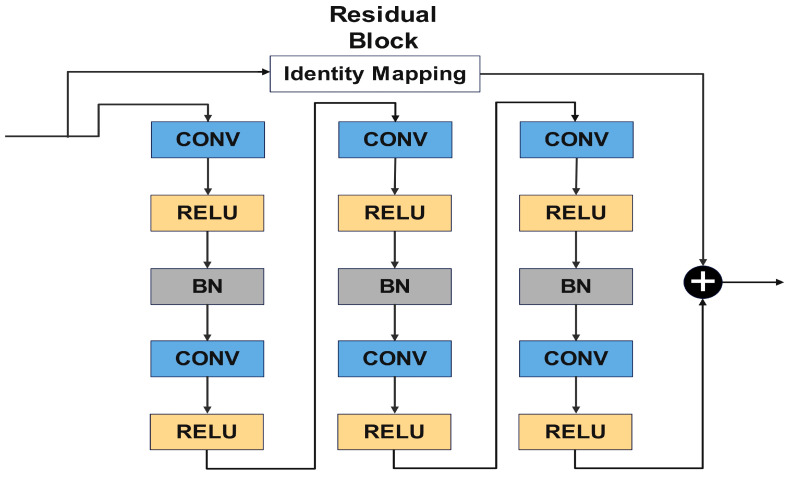
Structure of a residual block.

This connection allows the network to retain information from the original AlexNet layers while adding the transformations introduced by a residual block. The structure of the residual block illustrates the identity mapping that facilitates the addition of transformed outputs from the convolutional layers. It is shown in Figure 6.

The structure of Residual Block 2 is similar to that of Residual Block 1. However, the first convolutional layer of Residual Block 2 contains 384 filters with a filter size of 3 × 3, a stride of 1 × 1, and padding set to the same. In Residual Block 3, the first convolutional layer contains 384 filters with a filter size of 3 × 3, a stride of 1 × 1, and padding set to the same.

The modified AlexNet model with residual blocks offers several advantages over standard deep learning models commonly applied in industrial fault diagnosis. The inclusion of residual blocks improves gradient flow, enabling the network to train efficiently even with increased depth. This allows the model to capture both spatial and temporal features from CWT scalograms, which are essential for accurate fault classification. Additionally, the model significantly reduces the number of parameters, from 60 million in the original AlexNet to 9.8 million in the modified version. This makes it computationally efficient and suitable for real-time applications. The architecture was tailored to process CWT scalograms effectively, outperforming existing methods like standard CNNs and other reference models. Table 1 presents a structured breakdown of the various layers within the neural network, detailing the layer type, activation size, and learnable parameters. It starts with convolutional layers (Conv2D) using filters of different sizes (e.g., 3 × 3 and 5 × 55), and describes their corresponding output activation dimensions. Each layer is followed by ReLU activation, and non-linearity and BN layers that normalize the output across channels are applied. Residual connections, shown as additional layers, combine the outputs from different layers to improve learning. The table also lists the key details, such as the number of filters, the filter size, and whether padding is applied, giving an overview of the architecture’s structure and parameter flow.

Residual blocks were incorporated into the modified AlexNet architecture to address the challenges associated with vanishing gradients and to improve training stability in deeper networks. The shortcut connections in residual blocks allow gradients to bypass one or more layers, ensuring efficient backpropagation and facilitating the learning of identity mappings. This enables the network to effectively capture both spatial and temporal features from the CWT scalograms without the risk of the performance degradation typically seen in deeper architectures. The advantages of residual blocks, including improved gradient flow and training stability, are well-aligned with the high performance observed in this study.

### 2.4. Data Augmentation

Data augmentation plays an important role in enhancing the proposed approach by boosting the model’s generalization capabilities through increased dataset diversity. Techniques like rotation, scaling, and flipping are applied to the original CWT representations, generating a broader and more varied set of training examples [34]. These transformations mimic a range of real-world conditions, reducing the risk of the model overfitting to particular data patterns and enabling it to learn more robust and representative features. By enriching the dataset, data augmentation significantly strengthens the fault classification model’s performance, ensuring greater adaptability across diverse industrial scenarios. Table 2 below shows the detailed augmented dataset.

To address the limited sample size, data augmentation techniques, including rotation, scaling, and flipping, were applied to the CWT scalograms. These transformations generated diverse training samples, simulating various real-world conditions. By increasing the dataset size and variety, these techniques enabled the model to learn from a broader range of samples, improving its generalization capabilities and reducing the risk of overfitting.

### 2.5. Ant Colony Optimization

In nature, ants emit a pheromone trail as they travel between a food source and their colony. The more frequently a path is used, the higher the concentration of pheromones; thus, more ants follow that path. Over time, this collective behavior helps ants find the shortest and most efficient route. ACO mimics this behavior in feature selection by finding the most relevant features contributing to accurate fault classification [35].

Let F be the set of all features extracted from the modified AlexNet model, where the size of the feature set is 640 × 1000. The objective of ACO is to select a subset, Fopt⊂F, thereby reducing the feature set to 640 × 800 while retaining the features that provide the best discrimination between fault types.

Each feature $f_{i}\in F$ is represented as a node in the ACO search space. The ant traverses this space, selecting features to form a candidate solution (i.e., a subset of features). Let $S=\{f_{1},f_{2}\ldots .f_{m}\}$ represent the feature subset selected by an ant, where m≤n and n is the total number of features. The pheromone level determines the probability of selecting a feature $\tau_{i,j}$ and heuristic information $\eta_{i,j}$. The selection probability is given in Equation (7).

(7) $$P_{i,j}=\frac{\tau_{i,j}^{\alpha }\cdot \eta_{i,j}^{\beta }}{\sum_{k=1}^{n}\tau_{k,j}^{\alpha }\cdot \eta_{k,j}^{\beta }}$$

Here, α controls the influence of pheromone levels, β controls the influence of the heuristic factor, and $\eta_{i,j}$ is the heuristic value associated with the feature $f_{i}$. The selected feature subsets are evaluated based on their classification accuracy using an SVM, and the fitness of each subset is calculated. The fitness function *F*(*S*) can be defined as Equation (8):

(8) $$F\left(S\right)=1/E(S)$$

where E(S) denotes the classification error rate when using the feature subset S. After evaluation, the pheromone levels are updated. Features contributing to improved performance receive more pheromones, while others decay. The update rule is shown as Equation (9):

(9) $$\tau_{i,j}\left(t+1\right)=\left(1-\rho \right)\cdot \tau_{i,j}\left(t\right)+{∆\tau }_{i,j}$$

The process repeats until a stopping criterion is met, and the best feature subset is selected. ACO was employed for feature selection to identify the most discriminative features for fault classification. ACO is inspired by the natural behavior of ants in finding optimal paths, which allows it to evaluate and prioritize features based on their contribution to classification accuracy. This method retained the spatial and temporal characteristics useful for distinguishing fault patterns while reducing the dimensionality of the feature set. The ACO-selected features were particularly relevant for fault classification because the optimization process focused on maximizing the separability of fault categories. By preserving only the most informative features, ACO improved the model’s performance and computational efficiency, making it well-suited for real-time fault diagnosis applications. Table 3 shows the ACO parameters.

The proposed integration of deep learning with ACO demonstrates significant advantages in feature selection by identifying the most discriminative features for fault classification. While this study focuses on the benefits of ACO, other feature selection methods, such as Principal Component Analysis (PCA) and Recursive Feature Elimination (RFE), are commonly used in fault diagnosis. Compared to PCA, which reduces dimensionality by transforming features into orthogonal components, ACO directly optimizes features for classification relevance, preserving interpretability. Similarly, unlike RFE, which iteratively removes features to improve performance, ACO considers feature interdependencies, making it more suited for high-dimensional and complex datasets.

### 2.6. SVM

SVM is a powerful classification algorithm, particularly effective for high-dimensional datasets. In this study, the SVM classifier was employed with a Radial Basis Function (RBF) kernel to handle non-linear relationships in the data. The key parameters were carefully tuned: the kernel parameter (γ) was set to 0.01, the regularization parameter (C) to 10, and the tolerance (tol) to $10^{-3}$. To address the class imbalance, the class weight was set to balanced. Table 4 summarizes the selected SVM parameters and their values.

### 2.7. Uniform Manifold Approximation and Projection (UMAP)

UMAP is a powerful and efficient algorithm used for dimensionality reduction, particularly in high-dimensional datasets, such as those generated in AE signal analysis for pipeline health diagnosis. UMAP is conceptually rooted in manifold learning, which assumes that high-dimensional data exist on a low-dimensional manifold that can be approximated and mapped onto a lower-dimensional space. This approach helps capture the underlying structure of the data while reducing their complexity, allowing for easier visualization and pattern recognition.

The theoretical foundation of UMAP is built on concepts from Riemannian geometry and algebraic topology. Specifically, UMAP utilizes the mathematical framework of fuzzy simplicial sets and Laplacian eigenmaps to construct a topological representation of the data. In the case of AE signals, which often consist of complex, high-dimensional data points representing various aspects of pipeline conditions, UMAP helps reduce the dimensionality while preserving both local and global structures. This preservation is particularly important for analyzing transient events and signal features that might indicate anomalies or early signs of pipeline degradation.

UMAP works by constructing a weighted graph where each data point is connected to its nearest neighbors, reflecting the local structure of the manifold. The weight of the edges between points is determined using a Riemannian metric that approximates the geodesic distance on the manifold. The weight function is given by Equation (10):

(10) $$\omega \;\left(x_{i}\;,\;x_{j}\right)=\exp\left(-\frac{max(0,\;d\left(x_{i},\;x_{j}\right)-\rho_{i}}{\sigma_{i}}\right)$$

where $d\left(x_{i},x_{j}\right)$ represents the distance between points $x_{i}$ and $x_{j}$, $\rho_{i}$ is the distance to the nearest neighbor of $x_{i}$, and $\sigma_{i}$ is a scaling factor that normalizes the distances.

## 3. Experimental Setup and Data Acquisition

**Figure 7:**
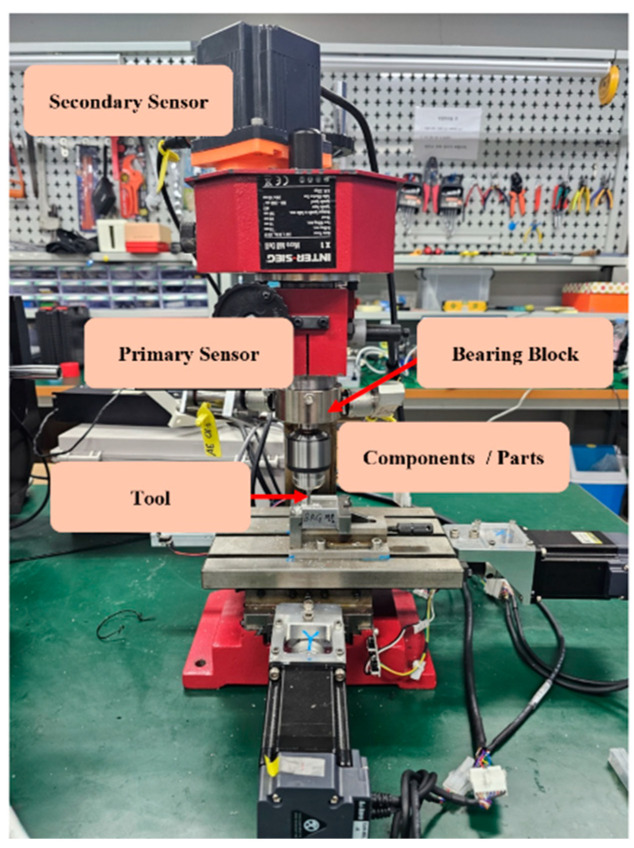
Experimental setup displaying the milling machine equipped with AE sensors.

**Figure 8:**
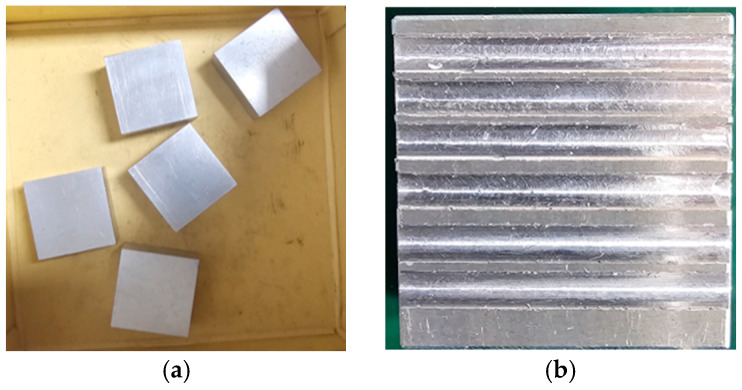
Examples of the materials used in the experiment: (**a**) raw workpieces; and (**b**) workpieces post-milling.

In the experiment of this study, AE signals were gathered from an operational milling machine, as shown in Figure 7. The milling procedures were carried out using an INTER-SIEG X1 Micro Mill Drill, a machine constructed from cast iron that functions similarly to a compact pillar drill. The operations focused on straight parallel milling on steel workpieces, a common technique for shaping and machining hard materials. For this experiment, five steel pieces were used, each measuring 20 mm, 35 mm, and 35 mm. Figure 8a illustrates the workpieces before processing, and Figure 8b displays one of the finished pieces after milling.

The two channels (channels 1 and 2) were set to identical acquisition conditions, including the same bandpass filter range and threshold settings, to ensure consistency across both channels. A bandpass filter was applied to each channel to remove low-frequency noise and high-frequency interference, which helped to optimize signal quality for subsequent analysis. Data from both channels were collected simultaneously to capture synchronous signals across different components, allowing for accurate, time-aligned comparisons in fault analysis. To monitor AE signals, the R15I-AST sensor from MISTRAS, Inc., USA, was affixed to the milling machine using industrial-strength adhesives. The AE data were acquired through the NI-9223 data acquisition module from National Instruments, with a custom software program written by the Ulsan Industrial Artificial Intelligence Laboratory in Python 3.11. The data were acquired at an ultra-high sampling rate of 1 MHz, with each second of data containing one million samples. Before actual data collection, the HSU-Nelson test was performed to validate the functionality of the AE sensors. The test successfully confirmed that both sensors were detecting AE events, thus ensuring they were ready for the experiment.

The experiment used two AE sensors: the primary sensor was attached to the spindle, while the secondary sensor, acting as a guard transducer, was mounted onto the motor. Data collection began under normal milling machine operation. According to the ISO-8688-2 guidelines, the tool lifespan is typically determined by an average flank wear of 0.3 mm. However, tools can sometimes fail unexpectedly early when machining hard materials. For this experiment, a carbide tool was thus artificially worn to an average of 0.3 mm, and AE data were collected under these defective conditions.

Additionally, an initial defect was introduced into the outer race of the bearing supporting the tool, and AE signals were recorded during the machining process. A minor metal fragment was also removed from one of the gear teeth that transmitted torque from the motor to the spindle, simulating a fault, and the corresponding AE signals were recorded during operation.

**Figure 9:**
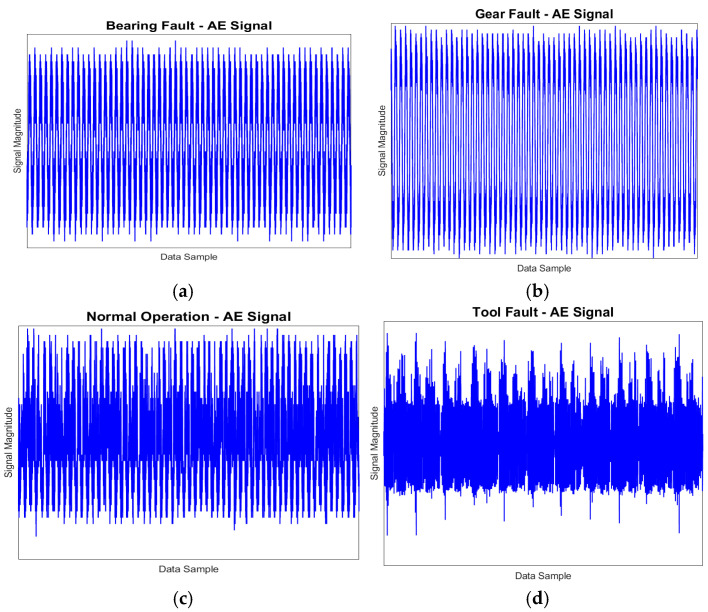
Fault diagnostics via AE time domain signals for various fault scenarios: (**a**) BF signal; (**b**) GF signal; (**c**) normal operation signal; and (**d**) TF signal.

**Figure 10:**
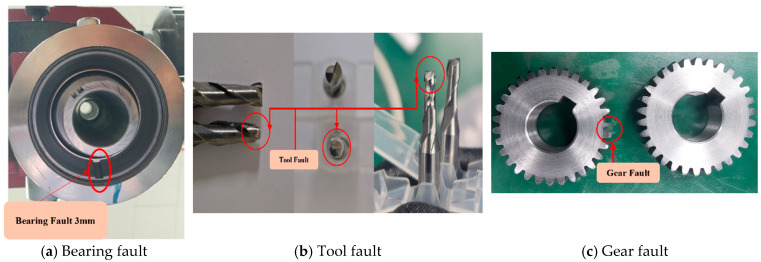
Components with induced faults used in the experimental setup: (**a**) bearing fault (BF); (**b**) tool fault (TF); and (**c**) gear fault (GF).

For each operating condition, a total of 40 samples were gathered. Table 5 presents a comprehensive summary of the dataset collected from the milling machine. To facilitate easier identification in the table, the normal operation condition is designated as “N”, while the faults related to the tool, bearing, and gear are labeled “TF”, “BF”, and “GF”, respectively. Examples of a 1-s AE signal recorded during N, TF, BF, and GF conditions are displayed in Figure 9. The faulty components used during the experiment are illustrated in Figure 10: the damaged bearing is shown in Figure 10a, the defective tool is depicted in Figure 10b, and the gear with faults is presented in Figure 10c.

## 4. Results

The creation of appropriate training and testing subsets was vital in assessing the effectiveness of the proposed approach in identifying the health condition of the milling machine. To this end, it was essential to create suitable training and testing subsets. This study used a dataset of 640 CWT images, with 160 images each representing the BF, TF, GF, and normal conditions. These images were then divided into training and testing sets to accurately assess the model’s performance.

### Performance and Comparison

In this study, we designed a method for fault detection using CWT scalogram images with the modified AlexNet architecture. The method is enhanced by data augmentation, contrast enhancement, and ACO for feature selection. The results showed superior performance compared to state-of-the-art reference models. The proposed approach involves data augmentation (rotation, scaling, flipping) to increase the dataset diversity and contrast enhancement to improve feature visibility. The modified AlexNet, with added residual blocks, efficiently captures the spatial and temporal features. ACO is used for feature selection, thereby reducing dimensionality while retaining key information and leading to precise classification with the SVM. In the experiment, various metrics, such as accuracy, precision, recall, F1 score, specificity, geometric mean, and computation time, were used to assess and compare the effectiveness of the proposed approach against established methods. Accordingly, Equations (11)–(16) are provided below, including the mathematical expressions used to calculate these metrics.

(11) $$Accuracy\frac{\left(TN+TP\right)}{\left(TN+TP+FN+FP\right)}\times 100\%$$

(12) $$Precision=\frac{TP}{\left(TP+FP\right)}\times 100\%$$

(13) $$Recall=\frac{TP}{\left(TP+FN\right)}\times 100\%$$

(14) $$F_{1}-Score=\frac{2TP}{2TP+FP+FN}=2\times \frac{Precision\times Recall}{Precission+Recall}$$

(15) $$Specificity=\frac{TN}{TN+FP}$$

(16) $$G-Mean=\sqrt{Recall\times Specificity}$$

In classification, TP (True Positive) refers to instances correctly identified as positive by the classifier, while TN (True Negative) refers to those correctly identified as negative. FP (False Positive) is a negative sample incorrectly classified as positive, and FN (False Negative) is a positive sample incorrectly classified as negative.

K-fold cross-validation can be employed to provide a more reliable estimate of model performance compared to a single training–test split. It reduces the risk of overfitting by testing the model on multiple independent subsets. In this work, five-fold cross-validation was employed as an effective evaluation strategy for diagnosing faults in the milling machine. The dataset was divided into five subsets, with the model trained on four and tested on each remaining one in each subsequent iteration. This process was repeated five times, ensuring each subset was used for testing once. The results were then averaged to provide an overall performance assessment. Regularization techniques were incorporated during model training to prevent overfitting. Dropout layers were added with a rate of 0.5, which temporarily deactivated 50% of the neurons during training to encourage the model to generalize better. Additionally, L2 regularization was applied with a penalty factor of 0.001, discouraging large weight values and ensuring a simpler model structure. These measures reduced the risk of overfitting while maintaining the model’s predictive performance.

Notable improvements were observed when applying the proposed technique to real-world AE data, with metrics such as the accuracy, precision, recall, F1 score, specificity, and geometric mean reaching values of 0.99, 0.9301, 0.9984, 0.9631, 0.9750, and 0.9866, respectively. Table 6 highlights the superiority of this approach over reference methods in terms of classification accuracy. The exceptional performance of the proposed method can be attributed to its fundamental concept of employing CWT-augmented scalograms, contrast enhancement, and the addition of residual blocks in the modified AlexNet, which enables the model to capture both spatial and temporal features more effectively. The process begins by acquiring CWT images for different fault and normal states. Data augmentation techniques are applied to expand the dataset, followed by contrast enhancement to improve feature visibility. The modified AlexNet architecture, enhanced with residual blocks, is then employed to extract dual temporal and spatial features from the augmented scalograms. After feature extraction, ACO is used to select the most relevant deep features. Finally, the SVM classifier is utilized to effectively classify the different fault classes, leading to efficient fault detection. The proposed approach provides superior time-frequency resolution, improved detection of transient events, reduced interference, and easier interpretability, which all contributed to its exceptional performance on all evaluation metrics. Additionally, it showed a lower computation time of 12.3 s, significantly outperforming the reference models in terms of efficiency.

**Figure 11:**
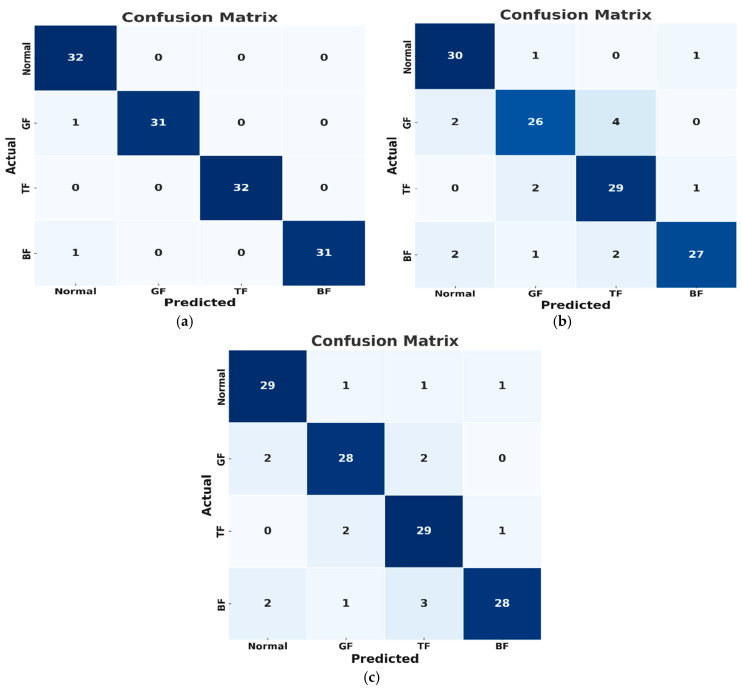
Confusion matrices for the (**a**) proposed model, (**b**) Weifang et al. [36] model and (**c**) CWT-CNN model.

The proposed model demonstrated substantial advantages in both methodology and performance metrics, as shown by the confusion matrices in Figure 11. To evaluate its efficacy, comparative analysis was performed with two other relevant models used for similar purposes. The first model, employed by Weifang et al. [36], employs a fault diagnosis approach based on converted 2D vibrational signal matrices. A mean curvature algorithm is applied to mitigate interference, and histograms of oriented gradient (HOG) features are employed for extracting fault characteristics. Moreover, an SVM is applied for automatic fault classification.

Implementing the steps outlined by Weifang et al. on our dataset resulted in an accuracy of 90.94%, precision of 73.39%, recall of 100%, F1 score of 84.66%, specificity of 87.92%, and geometric mean of 93.76%. The computation time for the model was 25.437 s. The underperformance and higher computation time were expected on account of the high noise levels affecting the AE signals. Moreover, the spectrograms lacked accurate energy distribution and failed to capture critical phase information. In contrast, the proposed model utilized CWT images, which provided better time-frequency resolution, improved transient event detection, reduced interference, and conferred easier interpretability.

**Figure 12:**
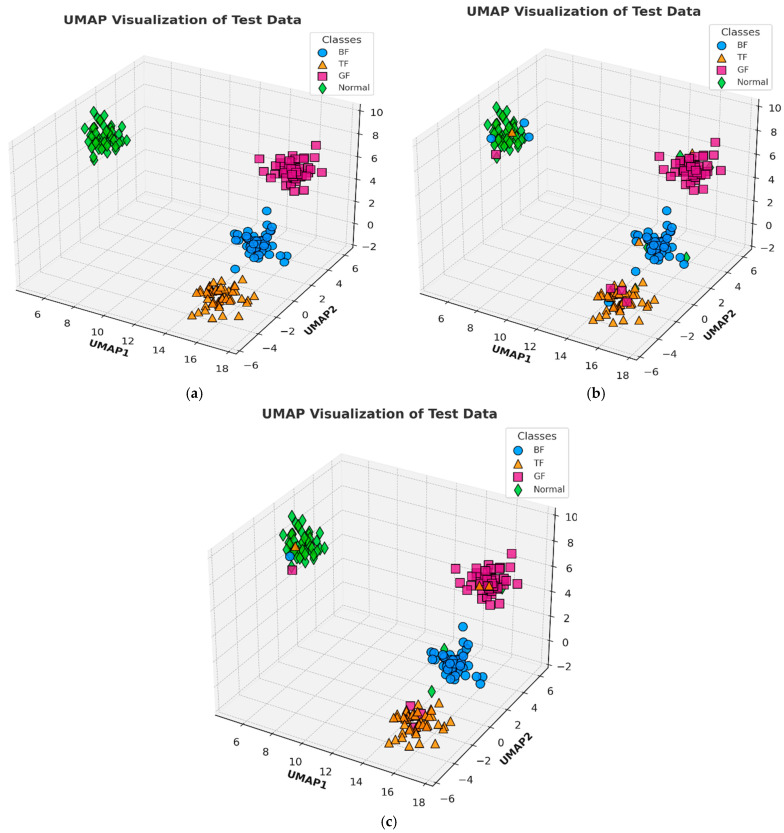
UMAP representation of the (**a**) proposed method, (**b**) Weifang et al. [36] method, and (**c**) CWT-CNN method.

The second compared method was the CWT-CNN approach, where AE signals are converted into CWT scalograms and then input into a CNN for feature extraction and classification. The CNN consists of convolutional, pooling, and flattened layers, followed by fully connected layers for fault classification. The CWT-CNN method achieved an accuracy of 93.52%, precision of 80.94%, recall of 96.88%, F1 score of 88.19%, specificity of 92.40%, geometric mean of 94.61%, and computation time of 24.963 s, as displayed in Table 6. While the high accuracy highlights the effectiveness of using CWT for extracting key features from AE signals, the method cannot explicitly capture temporal dependencies in the data. Figure 12 illustrates the UMAP representations for each method.

## 5. Conclusions

In this study, an approach for fault classification in milling machines using advanced image processing and machine learning techniques was designed. By acquiring raw data from real-world industrial environments and converting them into 2D CWT images, the model achieves superior time-frequency localization. Data augmentation and contrast enhancement are employed to improve feature visibility and dataset diversity, ultimately enhancing the model training. The modified AlexNet model, augmented with three residual blocks, proved effective in extracting both spatial and temporal features, which is crucial for understanding complex fault behaviors. Optimization using ACO enables dimensionality reduction while retaining key information, while the SVM classifier successfully differentiates fault types with high accuracy. The proposed method demonstrated outstanding performance across all metrics, achieving 99% accuracy, 93.01% precision, 99.84% recall, 96.31% F1 score, 97.50% specificity, and a geometric mean of 98.66%. Additionally, with a computation time of only 12.3 s, it offers a significant reduction compared to traditional approaches. These results validate the model’s high efficiency and effectiveness in accurately diagnosing faults. It is thus a promising solution for real-world industrial applications and has notable potential in predictive maintenance and improving the reliability of fault detection systems.

## Figures and Tables

**Table 1:** Modified AlexNet residual block layer architectures.

Layer Name	Layer Type	Activation Size (Spatial × Channels × Batch)	Learnable Parameters
Conv 1	Convolution	13 × 13 × 256 × 1	Weights: 3 × 3 × 256 × 2563 Bias: 1 × 256
ReLU 1	ReLU activation	13 × 13 × 256 × 1	-
BatchNorm 1	Batch normalization	13 × 13 × 256 × 1	Offset: 1 × 1 × 256 Scale: 1 × 1 × 256
Conv 2	Convolution	13 × 13 × 256 × 1	Weights: 5 × 5 × 256 × 256 Bias: 1 × 256
ReLU 2	ReLU activation	13 × 13 × 256 × 1	-
Addition 1	Addition	13 × 13 × 256 × 1	
Conv 3	Convolution	6 × 6 × 384 × 1	Weights: 3 × 3 × 256 × 384 Bias: 1 × 384
ReLU 3	ReLU activation	6 × 6 × 384 × 1	-
BatchNorm 2	Batch normalization	6 × 6 × 384 × 1	Weights: 3 × 3 × 256 × 384 Bias: 1 × 384
ReLU 3	ReLU activation	6 × 6 × 384 × 1	-
BatchNorm 2	Batch normalization	6 × 6 × 384 × 1	Offset: 1 × 1 × 384 Scale: 1 × 1 × 384
Conv 4	Convolution	6 × 6 × 384 × 1	Weights: 1 × 1 × 256 × 384 Bias: 1 × 384
ReLU 4	ReLU Activation	6 × 6 × 384 × 1	-
Addition 2	Addition	6 × 6 × 384 × 1	-
Conv 5	Convolution	2 × 2 × 384 × 1	Weights: 3 × 3 × 384 × 384 Bias: 1 × 384
ReLU 5	ReLU activation	2 × 2 × 384 × 1	-
BatchNorm 3	Batch normalization	2 × 2 × 384 × 1	Weights: 1 × 1 × 384 × 384 Bias: 1 × 384
ReLU 6	ReLU activation	2 × 2 × 384 × 1	-
Addition 3	Addition	2 × 2 × 384 × 1	-

**Table 2:** Detailed augmented dataset.

Condition Tested	Total Samples	Sampling Frequency	Time per Sample	Augmented Samples
Normal operation (N)	40	1 MHz	2 min	160
Tool fault (TF)	40	1 MHz	2 min	160
Bearing fault (BF)	40	1 MHz	2 min	160
Gear fault (GF)	40	1 MHz	2 min	160

**Table 3:** ACO parameters.

Parameter	Range	The Proposed Method Value Used
Number of ants ($N_{ants})$	10–15	20
Maximum iterations ($T_{max})$	50–200	100
Pheromone importance (α)	1–2	1.0
Visibility importance (β)	2–5	2.0
Evaporation rate (ρ)	0.1–1	0.5
Initial pheromone value ($\tau_{0})$	0.1–1	0.5
Selection strategy	Probabilistic	Probabilistic

**Table 4:** SVM parameters.

Parameter	Range	The Proposed Method Value Used
Kernel type	Linear, polynomial, RBF, sigmoid	RBF
Kernel parameter (γ)	$10^{-3}–10^{-1}$	0.01
Regularization parameter (C)	1–100	10
Tolerance (tol)	$10^{-4}–10^{-2}$	$10^{-3}$
Class weight	Balanced/none	Balanced

**Table 5:** Data collection overview.

Condition Tested	Total Samples	Sampling Frequency	Time per Sample
Normal operation (N)	40	1,000,000 Hz	2 min
Tool fault (TF)	40	1,000,000 Hz	2 min
Bearing fault (BF)	40	1,000,000 Hz	2 min
Gear fault (GF)	40	1,000,000 Hz	2 min

**Table 6:** Performance comparison of fault classification models.

Models	Accuracy	Precision	Recall	F1 Score	Specificity	Geometric Mean	Time (s)
Proposed model	0.99	0.9301	0.9984	0.9631	0.9750	0.9866	12.3
Weifang et al. [36]	0.9094	0.7339	1.0000	0.8466	0.8792	0.9376	25.437
CWT-CNN	0.9352	0.8094	0.9688	0.8819	0.9240	0.9461	24.963

## Data Availability

Data will be provided upon request.

## Nomenclature

AE	Acoustic Emission
CWT	Continuous Wavelet Transform
CNN	Convolutional Neural Network
SVM	Support Vector Machine
ACO	Ant Colony Optimization
EMD	Empirical Mode Decomposition
AEH	Acoustic Emission Hit
